# Co-Carriage of Metal and Antibiotic Resistance Genes in Sewage Associated Staphylococci

**DOI:** 10.3390/genes12101473

**Published:** 2021-09-23

**Authors:** Atena Amirsoleimani, Gail Brion, Patrice Francois

**Affiliations:** 1 Department of Civil Engineering, University of Kentucky, Lexington, KY 40506, USA; gail.brion@uky.edu; 2 Genomic Research Laboratory, Infectious Diseases Department, University Medical Center, 364-8501 Geneva, Switzerland; patrice.francois@genomic.ch

**Keywords:** *Staphylococcus aureus*, plasmid, wastewater, antibiotic resistance genes, metal resistance genes, gene content, gene transfer

## Abstract

Controlling spread of resistance genes from wastewater to aquatic systems requires more knowledge on how resistance genes are acquired and transmitted. Whole genomic sequences from sewage-associated staphylococcus isolates (20 *S. aureus*, 2 *Staphylococcus warneri*, and 2 *Staphylococcus delphini*) were analyzed for the presence of antibiotic resistance genes (ARGs) and metal resistance genes (MRGs). Plasmid sequences were identified in each isolate to investigate co-carriage of ARGs and MRGs within. BLASTN analysis showed that 67% of the isolates carried more than one ARG. The carriage of multiple plasmids was observed more in CC5 than CC8 *S. aureus* strains. Plasmid exchange was observed in all staphylococcus species except the two *S. delphini* isolates that carried multiple MRGs, no ARGs, and no plasmids. 85% of *S. aureus* isolates carried the *blaZ* gene, 76% co-carried *blaZ* with *cadD* and *cadX*, with 62% of these isolates carrying *blaZ*, *cadD*, and *cadX* on the same plasmid. The co-carriage of ARGs and MRGs in *S. warneri* isolates, and carriage of MRGs in *S. delphini*, without plasmids suggests non-conjugative transmission routes for gene acquisition. More studies are required that focus on the transduction and transformation routes of transmission to prevent interspecies exchange of ARGs and MRGs in sewage-associated systems.

## 1. Introduction

Antimicrobial resistance in bacteria is not a new phenomenon. Estimates for the emergence of biosynthetic pathways have been published that show this occurring hundreds of millions of years ago [1]. It has further been documented that bacteria have a pool of shared antimicrobial resistance genes (AMRGs) present in the antibiotic resistome prior to humans’ discovery and widespread use of antibiotics. In permafrost from 30,000 years ago, genes encoding resistance for β-lactam and other antibiotics have been documented [2]. There are self-transmissible plasmids carrying antibiotic resistance genes (ARGs) that can infect many phylogenetically distinct bacteria creating a pool of genes to share [3]. However, the role that human actions play in adding to the antibiotic resistome, and spurring a rapid increase of the numbers and diversity of antibiotic resistant bacteria (ARB), is relatively new ramping up with the discovery and wide-spread use of penicillin in the 1940′s. The number of daily antibiotic doses increased from 21.4 to 34.8 between 2000 to 2015, with a 40% daily dose rate increase per thousand inhabitants in 76 countries [4]. So, while antibiotic resistance is ancient, human activities have increased the numbers and types of antibiotic resistance strategies deployed by bacteria by loading the environment with antibiotics, creating conditions that apply pressure to clinical and environmental bacteria to adapt.

Human usage of antibiotics has resulted in the enhanced resistance of well-known, and opportunistic bacterial pathogens, to multiple antibiotics through mutation and acquisition of ARGs [1]. About 65% of these antibiotics are β lactam types and their usage continues to increase [5] Inputs from systems that grow livestock and treat sewage have made wastes generated from these activities the largest, continuous input of antimicrobials and antibiotics into aquatic systems, including the sediments and biofilms within [6,7]. With aquatic systems that serve as sinks for antibiotics and resistant organisms generated by human activities, the potential for gene transmission from human and animal pathogens, to environmental bacteria, then back to human and animal pathogens is probable [8,9]. Bacteria exchange genetic information horizontally through direct uptake of DNA (transformation), virally-mediated transduction, and pili-mediated conjugation. Sub-lethal levels of antibiotics and other antimicrobials, such as those found in sewage-associated aquatic systems, can stimulate horizontal gene transfer (HGT) and modulate gene transcription in bacteria, creating a flow of resistance genes between environmental and clinically significant bacteria [8,9]. The predominant direction of the flow is not well understood, but with other factors creating low fitness costs for the carriage of antimicrobial resistance genes (AMRGs), and documented transmission of these genes to environmental bacteria, aquatic systems are thought to be important, long-term reservoirs for antimicrobial resistance genes. To control the spread of AMRGs, the ways clinical and environmental bacteria share genetic materials must be understood.

ARGs often co-occur with biocidal/metals resistance genes (BMRGs) in mobile genetic elements [1] and are found in bacteria that are associated with human activity. A recent study of fully sequenced bacterial genomes and plasmids (*n* = 4582) has provided more insight into the co-occurrence of ARGs and BMRGs. In situations where the ARGs and BMRGs were located on the same plasmid, the plasmid size was larger (76 kb average) in comparison with plasmids that carried only ARGs [10]. The larger size of these plasmids was linked with the plasmids being conjugative, which would require close contact between the bacteria for HGT, while the larger size would incur survival costs to the bacteria to retain the plasmid. However, survival costs are lowered by increases in the levels of antimicrobials or metals in the aquatic matrix, and sewage has enhanced levels of both in comparison with natural aquatic systems. Sewage has antibiotics, disinfectants, cleaning compounds, metals, and other antimicrobials in solution, although in sublethal concentrations 100 times lower than that found in hospital sewage [6]. These concentrations are thought to be linked to an increase in AMRGs, ARGs, and BMRGs in the effluent bacteria that survive disinfection. It has been shown that while enterococci decreased in concentration by log factors through sewage treatment, these bacteria, 50% of which were antibiotic resistant, were still present in up to the thousands of CFU per mL of effluent [7]. A study reported that the final effluent of an urban water treatment plant could discharge 10^7^ to 10^10^ antibiotic resistant bacteria per inhabitant [7]. Clearly sewage effluent is a source of antibiotic resistance into our aquatic systems.

Staphylococci bacteria have been found in sewage and sewage effluents. Staphylococci are often isolated with several other species co-growing as skin commensals. Many species can cause infections of the skin or blood and organs, and a quick way to differentiate the members of this family is by coagulase testing. There are numerous types of coagulase-positive staphylococci (CoPS), from the well-known pathogenic, methicillin resistant *Staphylococcus aureus* (MRSA) of clinical significance to humans that has also been found in livestock, to *S. delphini*, a strain of veterinary concern that has recently had a documented human case reported [11]. There are clinically significant, coagulase-negative staphylococci (CoNS) as well: *S. epidermidis* and *S. warneri*, both commensal bacteria that can opportunistically cause device-associated infections, although more rarely for *S. warneri*. Of the CoNS skin commensals, *S. epidermidis* is isolated in greater numbers and more frequently from the skin than *S. warneri* [12], which comprises <1% of the total Staphylococcal skin flora [13]. Staphylococci have numerous mobile genetic elements, and readily exchange genetic materials through HGT. There is high homology between CoNS and CoPS *mecA* cassettes [14], and the cassette has been isolated from several species of staphylococci. Of the plasmids investigated for *S. aureus*, 70% had AMRGs with 20% carrying both ARGs and BMRGs [10].

The function of antibiotics and AMRGs in clinical settings changes after releasing them into the non-clinical environment [15]. Wastewater treatment plants are counted as a significant, anthropogenic source for spreading antibiotics, AMRGs, and antibiotic resistance bacteria by releasing their effluents into the natural waters [7]. The presence of *S. aureus* with the carriage of ARGs and MRGs has been reported in wastewater in multiple studies [16,17,18,19]. However, the possible mobile genetic elements (plasmids, transposons, bacteriophages, and integrons) for hosting resistance genes and potential pathways for exchanging, acquiring, and spreading them are vague among environmental staphylococci species [7]. Metagenomic studies have shown the presence of different resistance genes on sewage plasmids, and the significant role of these plasmids in transferring resistance genes through HGT [20]. It is necessary to identify the potential conditions that facilitate the acquisition of resistance genes of *S. aureus* origins to control their spread and retard future evolutions. Historically, plasmids were classified based on the type of replication (Rep) protein [21]; also named replicon [22]. Different selective pressures affect the diversity of plasmids. This means that the presence of a plasmid which encodes ARGs is relative to the clinical origins of the parent strain [23]. 

In this study, the prevalence and co-carriage of ARGs and MRGs were investigated in sewage-associated staphylococci species. Whole genome sequencing and BLASTN analysis was coupled to phenotypic screenings to detect plasmid-encoded resistance genes and determine bacterial origin of these mobile genetic elements in CoNS and CoPS. The growth of the more numerous *S. epidermidis* was suppressed to focus on potential gene sharing between identified strains of *S. aureus*, *S. warneri*, and *S. delphini* from isolates collected from sewage and sewage-impacted aquatic environments. Our prior research had shown the carriage of MRGs thought to originate in *S. aureus*, in *S. warneri*, and vice versa (results and data under review). It was also noted that *S. warneri* isolates were carrying MRGs originating from *S. delphini*. To further this work, we analyzed the ARGs in these isolates, and investigated if the ARGs (such as the *blaZ* gene) and MRGs (*cadD* and *cadX* genes) were carried in the sequences of identifiable plasmids, the plasmid partial or complete presence, and if the *blaZ*, *cadD*, and *cadX* were carried on plasmids in *S. aureus* and *S. warneri* or were present without being located in the identifiable plasmids.

## 2. Materials and Methods

### 2.1. Isolating and Sequencing of Staphylococci Isolates from Sewage-Impacted Environments

The sampling locations, events, selective enrichment media, identification and isolation procedures, DNA extraction, and whole genomic sequencing (WGS) methods used to obtain and type isolates of staphylococci from complex wastewater samples were completely described in the methods section of the previous published [17]. The location of each sampling site for collecting sediments in stream was summarized in the Supplemental Table S1. For sediment samples in sewage-impacted creeks, the applied method for wastewater samples was slightly modified to extract attached bacteria from sediments. Upon arrival of sediment samples to the lab, they were processed by mixing 60 g of the collected sediment samples with 200 mL of 1X mannitol salt broth. The mixture was shaken by hand for 10 min in a closed sterile bottle. Then, larger particles were settled out by gravity by resting the bottles on the bench for three minutes. After settling, the supernatant was slowly pipetted off and strained through a sterile, stainless-steel tea-strainer andsettled foranother three minutes before slowly pipetting 150 mL of the supernatant into a new sterile bottle. To this was added the same volume of 2X strength BBL^TM^ mannitol salt broth (Becton, Dickinson and company, Sparks, MD, USA) augmented with 0.0165 mg/mL acriflavine (Acrose Organics), 75 IU (international unit)/mL of polymyxin B (OXID), 30 mg/mL of nalidixic acid (Sigma Aldrich), 100 IU/mL Nystatin (Sigman Aldrich), and 3.5% potassium tellurite (OXID). After 24 h incubation at 37 °C, centrifugation was applied to 200 mL of the selectively cultured bacteria at 3700× *g* for 10 min in 225 mL sterile centrifuge tubes. The supernatant was removed andthe volume of the pellet was recorded and mixed with the same volume of 2X strength augmented selective broth and kept in incubator at 37 °C for 24 h. Another centrifugation with similar speed and time was applied to pellet bacteria prior to spread plating onto 1X strength mannitol salt agar plates augmented with similar concentrations of acriflavine, nalidixic acid, and polymyxin B as mentioned for the selective mannitol salt broth. Plates were kept in incubator at 37 °C for 48 h and then large yellow colonies were selected to streak plate 10 colonies, or 10% of all colonies, onto a new streak plate with selective agar. After another 48 h incubation at 37 °C, isolates on the streak plates were tested with three identification tests, OxiDrop^TM^ liquid (Hardy Diagnostic Z119), COAGULASE CRYO^TM^ (HARDY Diagnostic), and ELISA (MRSA latex test, Denka Seiken, Japan) as explained by Amirsoleimani et al., 2019. All selected isolates were inoculated into acriflavine-augmented BBL^TM^ mannitol salt broth, incubated at 37 °C for 48 h, and frozen at −80 °C until DNA extraction. Genomic DNA obtained from broth-cultured colony isolates was purified using the DNeasy kit (Qiagen, Courtaboeuf, France). DNA content of 50 μL extract aliquots was measured by Nano Drop, then extracts were shipped on ice to the University of Geneva Genomic Research Laboratory in Switzerland for whole genome sequencing. This subset was selected to avoid duplication of sampling days and provide wide-spread coverage of sampling sites.

Whole genomic sequencing was conducted on extracted isolate DNA run through an Illumina HiSeq 2500 (Illumina, San Diego, CA, USA) using 100 base reads with paired-ends according to the Truseq protocol (Illumina), following the manufacturer’s recommendations. The quality of sequence reads was assessed with the Fastqc program and reads were quality filtered using the fastq-mcf program. Genome assembly was performed using the Edena v3 assembler. Sequences (fasta files) of twenty *Staphylococcus aureus* isolates, two *Staphylococcus warneri* isolates, and two *Staphylococcus delphini* isolates were analyzed genetically by multiple tools available at the Centre for Genomic Epidemiology database, BLASTN, as well as available information at National Center for Biotechnology Information (NCBI) database. All isolate genomes have been deposited at ENA (https://www.ebi.ac.uk/ena, accessed on 12 August 2021) under the following name: PRJEB47642.

The *S. aureus* isolate genotypes were determined by multi-locus sequence type (MLST) number as developed by Enright et al. (2000) based on considering seven housekeeping genes in *S. aureus* isolates [24]. Each housekeeping gene was classified by a single number based on its sequence. Finally, a seven-digit number resulted from seven housekeeping genes identify the ST of one isolate.

### 2.2. Identification of MRGs and ARGs in Each Isolate

The database at the National Center for Biotechnology Information (NCBI) website and database (https://www.ncbi.nlm.nih.gov/ accessed on 30 April 2020) [25] was used to download the sequences for MRGs specific to *S. aureus*, *S. warneri*, and *S. delphini* on 30 April 2020. For each metal resistance gene selected, the BLASTN comparison was applied to compare the sequences of genes to make sure that the downloaded metal resistance genes did not have any similarity to each other. Then, the BLASTN analysis was used to compare the sequence of the non-similar downloaded genes to the fasta sequence of each bacterial isolate for the presence. For the comparison of the selected MRGs for cadmium, the percent of the query coverage (≥95%), percent identity (≥86%), and E values (≤1 × 10^−100^) were considered for identifying the presence of a specific MRG in the genome of the bacterial isolates (Supplemental Table S1). Different E cut-off values and percent identity have been considered for different studies and purposes. As an example Li et al. (2017) used E-values < 10^−^^5^ for determining that the alignment between two sequences was significant [26]. For percent similarity, Li et al. (2019) used 35% for proving similarity in their study [27]. However, for this study, we only accepted very low E values (<10^−100^) with high levels of coverage and identity to indicate carriage of MRGs with little change from the BLASTN sequences in our isolates.

In addition, the exact location of each gene on a specific contig was recorded based on the results of the BLASTN. This location and the context of the insertion of the gene was recorded for further analysis to see if the gene was encoded within a plasmid.

The resulting fasta files for the 24 sequenced isolates were further analyzed by the VirulenceFinder tool at the Centre for Genomic Epidemiology database (VirulenceFinder. https://cge.cbs.dtu.dk/services/VirulenceFinder/ accessed on 14 May 2020) [28]. This tool provided a summary related to all ARGs contained in the genome of one isolate with the exact location of the detected genes on a specific contig.

### 2.3. Plasmid Analysis

The presence of different plasmids in each bacterial isolate was investigated by applying the PlasmidFinder 2.1 tool at the Centre for Genomic Epidemiology database (https://cge.cbs.dtu.dk/services/PlasmidFinder/ accessed on 12 August 2021). This tool provided a list of different reference plasmids with their Accession numbers in each isolate. Based on the Accession number of an identified plasmidits, the fasta file was downlby by running BLASTN analyses the sequence of the reference plasmid was compared to the sequence of the uploaded isolate. The description table from the BLASTN analysis provided the exact location (the start and end point) of each plasmid, percent coverage between the sequence of the reference plasmid and the DNA sequence of an isolate, the query coverage, E value, and percent identity which was recorded for use in discussions about the level of similarity between the selected plasmids, and sequences under analysis. As stated prior, only very low E values were accepted for indicating the similarity of the plasmid sequence present in the isolates. In addition, the location of the plasmid in each isolate was recorded to compare against the location of the selected MRGs and ARGs on that plasmid for each isolate.

## 3. Results

### 3.1. Types of Isolates Based on Whole Genomic Sequencing

In this study, 24 sequenced sewage-associated staphylococci isolates were selected from all isolates archived to cover different sampling locations and different days. These 24 isolates were sequenced to investigate if MRGs and ARGs were present, and if they were located within plasmids. These isolates were obtained from sewage (influent, effluent, and sedimentation tank), or from a creek impacted by a wastewater treatment plant effluent (sediments from West Hickman creek), or where leaking sewers impacted sediment samples in a local park creek (Veterans Park creek-sediment). Among the 24 staphylococci isolates, twenty of them were identified as *S. aureus*, including 17 isolates found in wastewater (ID:1–18) and three isolates detected from creek sediment samples (ID: 51, 52, and 60). Two isolates were *S. warneri* (ID 54 and 55) isolated from the final chlorinated effluent, and two *S. delphini* isolates (ID: 53 and 56) were found in upstream sediment samples impacted by sewage leaks (ID:53), or downstream sediments impacted by the effluent of WWTP (ID:54) with one month differences in collection times. Classification of the *S. aureus* isolates showed the presence of three clonal complexes, CC59, CC5, CC8 with a number of different ARGs (Table 1).

Isolate #2 classified as CC59, which was the only isolate identified as the prevalent clonal complex in community isolates identified in Asiatic countries such as China, Taiwan, and Vietnam. It was recovered from a sewershed that had an airport, several hospitals, several college campuses, and high population density. Among the other of 19 *S. aureus* isolates, six of them typed as hospital-related *S. aureus* (CC5), and 13 isolates were classified as an environmentally-circulating strain of *S. aureus* (CC8). In this study all six isolates in CC5 subtyped as ST5. Isolates in CC8 had four different STs including ST8 (the ancestral genotype), ST2642 and ST1750 (single locus variants of ST8), and ST72 (triple locus variant).

### 3.2. Types of ARGs in Sewage-Associated Staphylococcus Isolates

Among the staphylococcus species in this study, five groups of antibiotic resistance phenotypes were detected as shown in Table 1 including β lactam, macrolide, streptogramin, aminoglycoside, and chloramphenicol antibiotics. As shown in Table 1, the *blaZ* gene was found in all *S. aureus* isolates except for three CC5-ST5 isolates (#13, 15, 18). All CC8 isolates carried the *blaZ* gene, but only 50% of CC5 isolates carried *blaZ* gene. The *mecA* gene was not as prevalent as the *blaZ* gene for β lactam resistance, with only 16% of CC5 (only 1 of 6 isolates) and 76% of CC8 (3 of 13) isolates positive. The *mecA* positive isolates were classified as methicillin-resistant *S. aureus* (MRSA) based on *mecA* presence. Based on these observations, it seems that the *blaZ* gene is well conserved in sewage-associated *S. aureus* and often associated with *mecA* in CC8 isolates. The presence of the *blaZ* gene may be related to the host type, and it is notable that both β lactam ARGs were absent in *S. warneri* and *S. delphini* isolates, which are animal associated.

For *S. aureus* CC8 isolates, only once was carriage of ARGs limited to a single gene (#8); and for those that had two ARGs, the second gene was most often *mecA*. Comparing the number of ARGs between different isolate types in CC8 shows that some strains carried more ARGs than others, with multiple types of antibiotic resistance common. In general, the average number of ARGs per *S. aureus* clonal complex, and the types of antibiotic class resistance, was higher in CC8-ST1750 (single locus variant of ST8) than for other *S. aureus* isolates, with isolate #3 carrying the maximum of six ARGs covering β lactam, macrolide, aminoglycoside, and chloramphenicol antibiotics. A second group of *S. aureus* isolates with higher numbers of ARGs was CC8-ST72, which is a triple locus variant of ST8 and frequently isolated in China and Korea and often harbouring numerous toxins [29]. Both isolate groups came from antibiotic-rich environments (WWTP influent or creek sediment under the influence of leaking sewers). It appears that within types of a clonal complexes, slight variations were associated with increased ARG carriage.

Isolates with only a single antibiotic-resistance gene were more prevalent in CC5 group (50%), and the resistance for macrolides more prevalent than for other antibiotic groups with, 66% of isolates positive for the presence of *erm(A)*, in contrast with 23% for all CC8 isolates. The other species of staphylococci had less ARGs present than *S. aureus*. Both *S. warneri* isolates (ID#: 54, 55) from fully treated WWTP effluent did not carry any β lactam antibiotic-resistance genes, but both isolates carried a unique type of antibiotic-resistance gene (*Vga(A)LC*) belonging to streptogramin phenotype, and one had an additional *erm(C)* resistance gene for macrolides. The *Vga(A)LC* gene was not found in the sequence of any other sewage-associated staphylococci isolates. The two *S. delphini* isolates from creek sediments were negative for the carriage of any ARGs.

Thus far, multiple research groups have reported the carriage of the *blaZ,* cadmium, and arsenic resistance genes on plasmids [30]. Therefore, the concurrence of these resistance genes was further investigated to illuminate possible associations between MRGs, ARGs, and plasmid transference in sewage-associated staphylococci isolates.

### 3.3. Types of Plasmids in Isolates

Table 2 summarizes the list of identified plasmids in this study with their accession numbers, lengths, rep numbers, and the specific bacterial types.

As shown in Table 2, the shortest plasmid in *S. aureus* group is the rep 10 plasmid (Accession #: GU562624) with 2402 base pairs, while the rest of plasmids in this group have lengths between 24,653 to 57,889. As shown in Table 2, plasmids were classified into three groups according to labelled species origination for reference plasmids specific to *S. aureus*, *S. warneri*, and *S. epidermidis*. The presence of selected plasmids, and the percent of the plasmid found in different isolates sequences are summarized in Table 3.

What is very clear is that *S. aureus* isolates carried a number of plasmids, but each clonal complex had differences in carriage, type, and number. The sewage-associated *S. aureus* isolates carried one to four plasmids, except for two isolates from CC5-ST5, (#13 from influent and #18 from effluent) that did not carry any plasmids, although they had shown carriage of *erm(A)* ARG. In general, CC8 isolates carried only single *S. aureus* plasmids, while CC5 carried multiple plasmids for 3 of the 4 plasmid carrying isolates.

There were some unique plasmid carriages associated with staphylococci species and *S. aureus* clonal complexes. Of the ten plasmids listed in Table 3, five of the plasmids originated, and were carried only in, a single species, or clonal complex of a species. The unique carriage of plasmids originating within a clonal complex of *S. aureus*, or a staphylococcus species, is indicative of the ability of these plasmids to be conserved within a group, with higher degrees of coverage indicating more recent acquisition and greater conservation. It is also suggestive of vertical gene transmission.

The rep 13 and 15 plasmids were found only in *S. aureus* CC5 isolates. The single *S. aureus* CC59 isolate (#2), carried a unique plasmid, the rep 5a, which was not found in any other isolates. The whole length of the rep5a plasmid was encoded in isolate #2 (100%). Both *S. warneri* isolates uniquely carried a plasmid labelled as originating in *S. warneri* (rep13a-AB125342) with 88% coverage as compared to the reference sequence.

Within the *S. aureus* isolates there were plasmids whose presence was not confined within a single clonal complex. The rep 19 plasmid was carried in one CC5 isolate (#14), and seven CC8 isolates, including four isolates of ST8 with only partial (52%) of sequence coverage, and three isolates of ST1750 with 52% and less coverage. The alignment results showed the exact same sequence of the partial 52% coverage of the rep 19 plasmid (8727 to 21,618 nucleotides) was encoded in all 4 isolates of ST8 and the single ST1750 sediment isolate (#51). Another shared within *S. aureus* species plasmid was the rep 20 (GQ900426). The rep 20 (GQ900426) plasmid was found in 50% of CC5 isolates and 23% of CC8 isolates, with varying degrees of coverage from 38% to 100%.

There were plasmids that appear to be shared between staphylococci species. *S. aureus* isolate #7 carried a *S. aureus* specific rep 10 plasmid at 100% coverage that was also found in one effluent *S. warneri* isolate sequence (#54). This *S. warneri* isolate (#54) contained 98% coverage of the sequence of the rep 10 plasmid in its genome. This was the only time that a plasmid (the rep10) was found both in the originating species, and a different staphylococci species in the isolates analysed. Some *S. aureus* isolates carried plasmids specific to other staphylococci species (*S. warneri* and *S. epidermidis*), but these plasmids were not present in the other species isolates that had been sequenced. In addition to the carriage of plasmids specific to *S. aureus*, isolate #1 (CC8-ST8) carried the rep13 plasmid specific to *S. warneri* with 42% query coverage, and over 99% identity, which means that 42% of this plasmid (E-value = 0) from another species was encoded with great accuracy in the sequence of isolate #1. In addition, *S. aureus* isolates #11 and #10 carried a partial sequence of the rep 20 plasmid specific to *S. epidermidis* with maximum carriage of 34% and 15%, respectively. Unlike isolates in the CC8 group, which captured some parts of plasmid’s sequences related to *S. warneri* and *S. epidermidis*, isolates belonging to CC5 group did not have any plasmids specific to coagulase-negative staphylococcus species.

Both S. *warneri* isolates (ID #54 and #55) isolated from final chlorinated effluent samples carried one plasmid specific to the coagulase-negative species *S. epidermidis*, the rep 5b plasmid with 58% query coverage. For both isolates the same part of the plasmid, 1956 to 5577 nucleotides, were encoded within the plasmid sequence and the sequence of two isolates were similar with E values equal to zero. While the presence of this partial plasmid is suggestive of horizontal gene transmission, the two isolates were found in effluent only a few days apart, and vertical gene transmission that retained the partial plasmid in the *S. warneri* that were able to persist in the treatment plant is also suspected.

The two, coagulase-positive *S. delphini* isolates were the only other type of CoPS species isolated in this study, and both were found in sediment samples from a sewage impacted creek at locations upstream and downstream of the WWTP with one-month difference in sampling. However, neither isolate carried any identifiable plasmids despite having three MRGs specific to *S. delphini* including *csoR* (copper-resistant), *arsB* and *arsC* (arsenic resistance) (data under review).

After identifying the unique and shared plasmids in each isolate, more analysis was conducted to try and link the presence of MRGs and ARGs to carriage of these plasmids.

### 3.4. Carriage of MRGs and ARGs on Plasmids

The results for the carriage of MRGs and ARGs on identified plasmids is summarized in Table 4. The two *S. delphini* isolates were not included in this table since they did not have any plasmids identified on their genome, although as mentioned they carried multiple MRGs specific to *S. delphini* (results did not present here).

The *blaZ* gene was the most prevalent ARG in the *S. aureus* isolates, but was not always carried within a plasmid. As shown in Table 4, 85% (17 out of 20) of the *S. aureus* isolates were positive for carrying the *blaZ* gene in their sequence, with 100% of the CC8 isolates positive. Among these *blaZ* positive isolates, 65% (11 out of 17) contained the *blaZ* gene within a plasmid. All 3 genes (*blaZ*, *cadD*, and *cadX*) co-occurred 13 times (two last columns in Table 4), with co-location on a plasmid detected 8 times (62%). *S. aureus* isolates were also carrying the *S. aureus* reference *cadD* and *cadX* MRGs within the same plasmid, but not co-located with *blaZ*. Three *S. aureus* isolates (#9, #12, and #3) with the *blaZ* gene did not carry any MRGs on their plasmid. The carriage of the *blaZ*, *cadD*, and *cadX* within the same plasmid was more common in CC8 isolates (5 out of 13 isolates, 38%) in comparison with CC5 (1 out of 6 isolates, 16%). The order of these ARGs and MRGs carriage was not consistent in all isolates. As shown in this table, in some isolates (#7, #6, #16, and #51) the *blaZ* gene was encoded after the cadmium resistance genes. However, in isolates #11 and #5, the *blaZ* gene was encoded before cadmium resistance genes.

The unique plasmid, rep 5a, in the single *S. aureus* CC59 isolate #2 encoded arsenic and cadmium resistance genes with the *blaZ* gene within its sequence. The antibiotic resistant gene *erm(C)* in isolate #7 was encoded on the rep 10 plasmid, not with the cadmium resistance genes. This finding supports previous research that has related the association of the *erm(C)* to the rep 10 plasmid [31].

Both *blaZ*-negative *S. warneri* isolates, ID# 54 and 55, isolated from the effluent of a WWTP were positive for 3 and 2 different plasmids, respectively. However, the metal (*S. aureus cadD* and *cadX*) and antibiotic resistance (*erm(C)* and *Vga(A)LC*) genes were not carried within the identified plasmid sequences. This is indicative of the acquisition of ARGs and MRGs specific to *S. aureus*, and carried on *S. aureus* plasmids, by *S. warneri* isolates by other means than directly exchanging *S. aureus* specific plasmids by conjugation.

## 4. Discussion

A plasmid analysis of sewage-associated staphylococcus species was conducted in this study to investigate if MRGs and ARGs encoded on plasmids were carried by more than one species, or clonally related to a species. In addition, the co-location of ARGs and MRGs on plasmids was investigated to see if HGT between species, and strains of species, was likely by conjugation. Understanding the relationship between the reference plasmids and encoded MRGs and ARGs is important to comprehend how these resistance genes may spread in the sewage-associated environment. We showed that the presence of different plasmids is related to the lineage of *S. aureus* since rep19 and 20 were found in most of isolates in CC8 and CC5 groups. These two clonal complexes are widely distributed in human and veterinary medicines. Different plasmid groups of *S. aureus* isolates were mentioned by McCarthy and Lindsay (28) to show what MRGs, ARGs, and virulent genes are encoded on different plasmids in each group. Based on their findings, the *blaZ* and the *cadDX* genes are two variable resistance genes (they are not core genes but transferred by mobile genetic elements) that occur together on the rep 19 plasmid [31]. The linked carriage of the *blaZ* and cadmium resistance genes (*cadD* and *cadX*) was shown in another study [32] and our results support both of these studies findings as our observations also related the encoding of the *cadD*, *cadX* and *blaZ* genes together on the same plasmid. These markers are quite abundant in strains isolated from environmental samples and are not restricted to Gram-positive bacteria but are also present in several Gram-negative species [33,34,35].

The McCarthy and Lindsay (2012) study documented the presence of rep 5 in *S. aureus* CC15, CC25, CC30, and CC45 [31]. Our findings expand theirs by documenting the rep 5 plasmid in CC59, a group of community *S. aureus* strains prevalent in numerous Asian countries. This difference in our findings could be related to the different environment (country-origin) that their isolates came from, or to a more recent acquisition of rep 5a by the CC59 isolate in our study. Also, as they noted the presence of the *blaZ* gene in all human lineages of *S. aureus* was documented, yet was rare among animal-associated *S. aureus* [31]. In this study, all *S. aureus* isolates except three CC5-ST5 isolates were positive for the presence of the *blaZ* gene. This is suggestive that our sewage-associated *S. aureus* isolates came predominately from human hosts where co-growth in skin niches could encourage HGT by conjugation, while stressors in this environment could encourage vertical transmission after gene acquisition.

The co-carriage of *blaZ* and cadmium MRGs commonly coded within *S. aureus* plasmids can be related to the environmental conditions around the bacteria that impact gene transfer. These encoded resistance genes on plasmids are exchanged via HGT with other *S. aureus* and staphylococci bacteria. The exchange of large, complete plasmids between two bacteria by conjugation are favoured in high-density conditions when bacteria have a direct contact to each other. That plasmid conjugation occurred can be inferred from our study results where a number of isolates contained large plasmids (rep 19 and 20) present in *S. aureus* at good levels of coverage, and had the *blaZ*, *cadD*, and *cadX* resistance genes located within those plasmids. However, the presence of these plasmids, even at greater coverages, did not assure the co-carriage of *blaZ* and cadmium resistance genes within the plasmid. The presence of rep 20 plasmids at 100% coverage in *S. aureus* CC8- ST2642 isolates #9 and #12 did not have the MRGs encoded within the plasmid, even though these genes were present in the isolate. So, the presence of the rep 19 and 20 plasmid was not always associated with the presence of the *blaZ* and cadmium resistance genes within the plasmid.

In this study, 35% (7 out of 20) of *S. aureus* isolates carried the *blaZ* gene with *cadD* and *cadX* gene on the same plasmid, one isolate carrying these genes in two separate plasmids. The plasmid most frequently carrying all three genes was the rep 19, which occurred 6 of the 8 times. If we consider all *S. aureus* isolates that carried ARGs, the co-carriage of both cadmium resistance genes and ARGs in a plasmid sequence occurred in 40% (8 of 20) of the *S. aureus* isolates in this study. This percentage of co-occurrence of ARGs and MRGs is higher than reported by Pal et al. (9) for 4582 plasmid sequences from publicly available bacterial genomes, only 5% of plasmids carried both ARGs and BMRGs. They documented 20% of co-occurrence of ARGs and BMRGs on plasmids for Staphylococcus genomes (9), which is less than half the frequency we found for our isolates, even though we selected for only cadmium-specific MRGs instead of any AMRGs co-occurrence with ARGs. It would seem that there is more research needed on the co-carriage on ARGs and AMRGs in sewage-associated environments to help understand the changes in the resistome present in polluted aquatic systems.

For other sewage-associated staphylococci species, the lack of plasmids and presence of ARGs and MRGs outside of plasmids, suggests that the acquisition of genes from sewage-associated *S. aureus* can occur by other gene transfer mechanisms. As mentioned by Pal et al. [10], the frequency of co-carriage of ARGs and BMRGs for the bacteria they examined was greater in larger plasmids, with a median size of 76 kb. In our study, the size of plasmids (rep 19, 20, and 5a) carrying *blaZ*, *cadD*, and *cadX* were smaller than 30.4 kb, and even smaller when taking into consideration that often only partial plasmids were found (<53% coverage rep 19). Their study did show a number of ARGS and AMRGs in plasmids of 25kb average size, but none in plasmids less than 20 kb (9), suggesting that the mode of gene transfer for these smaller, partial plasmids, was not conjugative.

For the two *S. warneri* isolates, conjugation of plasmids does not appear to be the primary mode for acquisition of *S. aureus* reference cadmium MRGs, nor macrolide and streptogramin B ARG acquisition since neither of these isolates had any plasmids identified that carried the genes. This lack of plasmid co-carriage of ARGs and MRGs could be related to its hosts and prevalence in the broader environment. *S. warneri* are known to inhabit animal hosts [36] as well as humans, and have been implicated in human disease [37]. However, even though *S. warneri* may grow in co-culture on animal hosts in small numbers, in sewage-associated aquatic systems, we did not find co-carriage of MRGs and ARGs on plasmids, suggesting another mechanism for HGT acquisition of these genes from other species of bacteria.

*S. warneri* can co-grow on human hosts as it has been reported to be the next most frequently identified, coagulase-negative, methicillin resistant, staphylococcus species after *S. epidermidis* found colonizing baby-care personnel and is known to carry the SCC cassette that encodes for *mecA* [38,39,40]. However, *S. warneri* is not as commonly found on humans as other staphylococci, often found on animals, and our study found a lesser prevalence in untreated sewage than *S. aureus*. Only in the treated sewage effluent, after chlorination, was *S. warneri* the dominant species consistently isolated from samples. *S. aureus* was isolated by selective growth enrichment from effluent only once in two years of sampling by comparison. It is unknown if S. *warneri* can grow inside the sewage treatment plant, but the continuous presence in the treatment plant effluent after chlorination points to an ability to survive, and perhaps even grow in these conditions.

In the effluent environment, after sedimentation has cleared microbially dense sludge biofilms from the water column resulting in lower bacterial densities, genetic information can still be transferred by transduction, using unsettleable bacteriophage as vectors between bacteria, or by transformation. Generalized transduction could result in the uptake of pieces of genetic material from other bacteria in the DNA and bacteriophage-loaded effluent, leading to the acquisition of genes without the acquisition of complete plasmids, especially in the presence of different environmental stresses such as toxic compounds or biocidal molecules [38]. As mentioned by Pal et al. [10], HGT of ARGs is increased by exposure to metals and biocides, and they suggested that use of zinc and cadmium supplementation could potentially promote co-selection for, and HGT of, ARGs and BMRGs. In our study results, we found cadmium resistance reference gene sequences for *S. aureus* in *S. warneri*, which suggests HGT between these staphylococci species. However, the presence of reference *S. aureus* cadmium resistance genes and absence of *blaZ*, without the presence of the rep 19 and 20 plasmids known to encode these resistance genes together, along with the presence of *erm(C)* without the rep 10 plasmid, suggests that the two *S. warneri* isolates acquired MRGs and ARGs via HGT by transduction, or direct DNA uptake, from non-viable *S. aureus*.

The acquisition of resistance genes from other staphylococci associated with escalating disease burdens, into *S. warneri* that are being consistently released into the environment from our WWTPs in large numbers, is concerning and potentially hazardous to animals and people encountering ARB in receiving waters. *S. warneri* has been shown by others to carry *mecA* and have methicillin resistance. The acquisition of plasmids specific to *S. aureus* (the rep 10) and *S. epidermidis* (the rep 5b) provide more confirmation about the ability of *S. warneri* to obtain, carry, and spread plasmid-associated resistance genes from organisms shed from human hosts into aquatic systems via transport by WWTP effluent into receiving streams. As WWTP effluent can provide a significant source of water in receiving streams, especially in arid regions, the potential for *S. warneri* to obtain virulence genes and pass them to other bacteria needs to be researched so this risk can be fully understood.

Our results showed how cadmium resistance genes were shared between *S. aureus* and *S. warneri* sewage isolates, but did not show that these other staphylococci were capable of sharing genes with *S. delphini* isolates present in the receiving creek sediments. The environmental conditions, or the resistance of *S. delphini*, seems to be preventing gene transference, as indicated by the lack of plasmids, ARGs, and MRGs from other staphylococci bacteria in both *S. delphini* isolates. The analysis of MRGs (data did not presented here) showed that these two *S. delphini* isolates did not carry any MRGs specific to *S. aureus* or *S. warneri*. However, MRGs specific to *S. delphini* were found in both *S. delphini* isolates, and other staphylococcus species isolates in this study, suggesting that while HGT between species can occur, it is limited due to differences in the staphylococci species ability to acquire and spread virulence factors from human pathogens to other bacteria. A better understanding of the resistance of *S. delphini* to HGT may help us lessen the transference in other staphylococci.

ARGs carriage was variable among the *S. aureus* isolates by type. Macrolide resistance was detected in a greater percentage in CC5 isolates in this study, which was different from reported results by McCarthy and Lindsey [31] since the presence of the *erm(A)* gene was reported in CC8 and CC239 lineages and not in CC5 in their study. Macrolide resistance was found to be more prevalent in CC8 isolates, but not in the ST8 group of isolates.

Our results showed that isolates from two types of *S. aureus*, CC8-ST 1750 (single locus variant of ST8) and CC8-ST72 (triple locus variant of ST8), carried more ARGs from isolates found in wastewater and sediment in comparison with other CC8 types. This could be related to their emergence as human pathogens, with more host carriage, or to the evolution of greater survival in the antibiotic-loaded environment of sewage [7].

## 5. Conclusions

More research is required to investigate the interspecies exchange of AMRGs, ARGs, MRGs, and plasmids to detect possible pathways and engineer solutions that hinder gene transfer. Thus far, all WWTP studies have focused on detection of pathogenic MRSA being present at the end of the WWTPs. However, our research highlights the need to consider other more treatment-resistant microorganisms that can survive chlorination and have the potential to transfer ARGs and MRGs from clinically relevant bacteria out into the environment, like *S. warneri*. Our results demonstrate a need to revisit and update indicator bacteria regulations and standards regarding the quality treated sewage at the end of WWTPs prior to discharge into natural streams. This is crucial to protect users of waterways from unwarranted disease, especially infections related to *S. warneri*, during contact recreation. Research into the potential health impacts of bacteria that exit WWTPs is important to change treatment plant operations to minimize not only frank pathogens, but other bacteria and bacteriophages that can carry virulence factors and resistance genes into the stream environment. The encoding of multiple MRGs, plasmids specific to other staphylococci, and the carriage of ARGs not on the plasmid sequence by *S. warneri* is indicative that routes of gene uptake other than conjugation in sewage environments are more likely. Since, we showed that the acquisition of ARGs and MRGs is possible with bacteriophages, more studies are required to investigate the transduction and phage transmission of ARGs, MRGs, and other virulent genes at the end of WWTPs between bacterial species.

## Figures and Tables

**Table 1:** Type of antibiotic resistance genes in sewage-associated staphylococcus species.

Sample			Type			Antibiotic Resistance Genes and Phenotypes																
Type	ID	Location	Bacteria	CC ^13^	ST ^15^	ꞵ-lactam				Macrolide					Strep ^16^ B	Aminogly ^17^				Chloram ^18^		Total ARGs
						*blaZ*	*mecA*	*blaTEM-116*	*blaTEM-210*	*erm(A)*	*erm(C)*	*msr (A)*	*mphC*	*Isa(A)*	*Vga(A)LC*	*aph(3)-III*	*ant(6)-Ia*	*ant(9)-Ia*	*aac(6)-aph(2)*	*Cat(pC194)*	*fexA*	
ww ^1^	2	TB ^3^-Inf ^4^	SA ^10^	59	59	+																1
ww	7	WH ^5^-SediT ^6^	SA	5	59	+	+				+										+	4
ww	4	WH-Inf	SA	5	5	+																1
ww	14	WH-Inf	SA	5	5	+				+		+				+			+			5
ww	13	TB-Inf	SA	5	5					+												1
ww	15	WH-Inf	SA	5	5					+									+			2
ww	18	WH-Eff ^7^	SA	5	5					+												1
ww	8	WH-SediT	SA	8	8	+																1
ww	1	TB-Inf	SA	8	8	+	+															2
ww	6	WH-Inf	SA	8	8	+	+															2
ww	11	WH-Inf	SA	8	8	+	+						+			+						4
ww	16	WH-SediT	SA	8	8	+	+															2
ww	9	WH-Inf	SA	8	2642	+	+															2
ww	12	WH-Inf	SA	8	2642	+	+															2
ww	3	TB-Inf	SA	8	1750	+	+	+				+					+			+		6
ww	5	WH-Inf	SA	8	1750	+	+			+		+				+						5
sedi ^2^	51	VPC ^8^	SA	8	1750	+	+		+													3
ww	10	WH-Inf	SA	8	72	+	+						+				+					4
sedi	52	VPC	SA	8	72	+				+								+				3
sedi	60	VPC	SA	8	72	+				+												2
ww	54	WH-Eff	SW ^11^	N/A ^14^	N/A						+				+							2
ww	55	WH-Eff	SW	N/A	N/A										+							1
sedi	53	VPC	SD ^12^	N/A	N/A																	0
sedi	56	WHC ^9^	SD	N/A	N/A																	0

^1^ Wastewater|^2^ Sediment|^3^ Town Branch|^4^ Influent|^5^ West Hickman|^6^ Sedimentation Tank|^7^ Effluent|^8^ Veterans Park Creek|^9^ West Hickman Creek|^10^ *S. aureus*|^11^ *S. warneri*|^12^ *S. delphini*|^13^ Clonal Complex|^14^ Not Applicable|^15^ Sequence type|^16^ Streptogramin|^17^ Aminoglycoside|^18^ Chloramphenicol.

**Table 2:** List of considered plasmids in this study.

Accession #	Specific to	Rep #	Plasmid Length (bp)
AP003139	*S. aureus*	5a	24,653
GU562624		10	2402
AF051917		13	46,445
AE017171		15	57,889
CP000704		19	3,0429
GQ900426		20	27,268
AM040729	*S. warneri*	13	5767
AB125342		13a	2779
GQ900459	*S. epidermidis*	5b	6206
GQ900453		20	16,775

**Table 3:** Plasmid in each staphylococci isolate with percent of query coverage (top value) and percent identify (bottom value).

Plasmids Based on Bacterial Types and Rep #														
Specific to Bacterial Type →					*S. aureus*						*S. warneri*		*S. epidermidis*	
Isolate ID	Bacterial Type	CC		Rep # →	5a	10	13	15	19	20	13	13a	5b	20
			St											
2	*S. aureus*	59	59		100 99.81				48 98.75					
7	*S. aureus*	5	5			100 99.77				99 99.90				
4	*S. aureus*	5	5							49 99.95				
14	*S. aureus*	5	5				40 99.98	31 99.99	32 99.96	75 99.65				
15	*S. aureus*	5	5				42 99.86	31 99.99						
13	*S. aureus*	5	5											
18	*S. aureus*	5	5											
8	*S. aureus*	8	8											
1	*S. aureus*	8	8						52 99.36		42 99.64			
6	*S. aureus*	8	8						52 99.35					
11	*S. aureus*	8	8						52 99.33					34 98.35
16	*S. aureus*	8	8						52 99.35					
9	*S. aureus*	8	2642							100 99.87				
12	*S. aureus*	8	2642							100 99.87				
3	*S. aureus*	8	1750						15 98.14	38 99.96				
5	*S. aureus*	8	1750						26 98.89					
51	*S. aureus*	8	1750						52 99.35					
10	*S. aureus*	8	72											15 98.82
52	*S. aureus*	8	72											
60	*S. aureus*	8	72											
54	*S. warneri*	N/A ^1^	N/A			98 99.53						88 99.33	58 90.97	
55	*S. warneri*	N/A	N/A									88 99.23	58 90.97	
53	*S. delphini*	N/A	N/A											
56	*S. delphini*	N/A	N/A											

^1^ Not applicable; Note: All E-values were zero.

**Table 4:** The presence of metal and antibiotic resistance genes on plasmids in sewage-associated staphylococci species (values in two last columns: percent query coverage (top), E-value (middle), and percent identity (bottom).

Isolate				Plasmid		*blaZ*	MRGs & ARGs on the Plasmid with Respect to Their Order on a Contig	BLASTN Results	
						Accession # CP000731		SA-*cadD*	SA-*cadX*
Type	ID	CC ^3^	ST ^4^	Rep #	Specific to			Accession #: WP_012818129.1	Accession #: WP_000119677.1
WW ^1^	2	59	59	19	SA ^6^	+	*blaZ→ cadX→ cad D*	100 * 0 ** 92.56 ***	100 7 × 10^−164^ 96.26
				5a			*arcC→ arsB→ arsR→ blaZ→ cadX→ cadD*		
WW	7	5	5	20	SA	+	*cadD→ cadX→ blaZ*	100 0 96.26	100 7 × 10^−164^ 96.26
				10			*erm(C)*		
WW	4	5	5	20	SA	+	*cadX→ cadD*	100 0 92.23	100 7 × 10^−164^ 96.26
WW	14	5	5	19	SA	+	*cadX→ cadD→ msr(A)*	100 0 92.56	100 7 × 10^−164^ 96.26
WW	15	5	5		SA				
WW	13	5	5		SA				
WW	18	5	5		SA				
WW	8	8	8			+			
WW	1	8	8	19	SA	+	*cadD→ cadX*	100 0 92.56	100 7 × 10^−164^ 96.26
WW	6	8	8	19	SA	+	*cadD→ cadX→ blaZ*	100 0 92.56	100 7 × 10^−164^ 96.26
WW	11	8	8	19&20	SA	+	*blaZ→ cadX→ cadD*	100 0 92.56	100 7 × 10^−164^ 96.26
WW	16	8	8	19	SA	+	*cadD→ cadX→ blaZ*	100 0 92.56	100 7 × 10^−164^ 96.26
WW	9	8	2642	20	SA	+	*blaZ*	100 0 92.23	100 7 × 10^−164^ 96.26
WW	12	8	2642	20	SA	+	*blaZ*	100 0 92.23	100 7 × 10^−164^ 96.26
WW	3	8	1750	19&20	SA	+	*blaZ*	100 0 92.23	100 8 × 10^−164^ 96.26
WW	5	8	1750	19	SA	+	*blaZ→ cadX→ cadD*	100 0 92.56	100 8 × 10^−164^ 96.26
Sedi	51	8	1750	19	SA	+	*cadX→ cadD→ blaZ*	100 0 92.56	100 7 × 10^−164^ 96.26
WW	10	8	72	20	SA	+			
Sedi	52	8	72			+			
Sedi ^2^	60	8	72	20	SA	+	*cadX*		96 4 × 10^−104^ 86.65
				7c	SA		*arsR→ arsB*		
WW	54	N/A ^5^		10	SA		No co-carriage of MRGs and ARGs on plasmids	100 0 92.12	100 3 × 10^−160^ 95.69
				13	SW ^7^				
				5b	SE ^8^				
WW	55	N/A		13	SW		No co-carriage of MRGs and ARGs on plasmids	100 0 96.12	100 1 × 10^−160^ 95.69
				5b	SE				

^1^ Wastewater|^2^ Sediment|^3^ Clonal Complex|^4^ Sequence Type|^5^ Not applicable|^6^ *S. aureus*|^7^ *S. warneri*|^8^ *S. epidermidis*|* Query coverage (%)|** E value|*** Percent identity (%).

## Supplementary Materials

The following are available online at https://www.mdpi.com/article/10.3390/genes12101473/s1, Table S1: Sediment sampling locations.
