# Supplementary material for: Co-Carriage of Metal and Antibiotic Resistance Genes in Sewage Associated Staphylococci
Source: Genes (Basel). 2021 Sep 23;12(10):1473. doi: 10.3390/genes12101473 (PMC8535820; doi:10.3390/genes12101473)
Supplement: Supplementary file 1 [file genes-12-01473-s001.zip › genes-1359671-supplementary.pdf]

Table S1: Sediment sampling locations

**Sample Location ID**	Sed-Stream-1	Sed-Stream-2	Sed-Stream-3	Sed-Stream-4
**Location**	Small stream upstream of Veterans Park	West Hickman Creek (upstream of Veterans Park)	Veterans Park Creek	West Hickman Creek (downstream of WWTP*)
**Latitude**	37° 58′ 49.92” N	37° 58′ 50.22” N	37° 57′ 29.08” N	37° 56′ 4.61” N
**Longitude**	84° 29′ 26.09” W	84° 29′ 25.15” W	84° 30′ 7.09” W	84° 30′ 7.44” W

*Wastewater Treatment Plant
